# Dental Panoramic Radiographs as Opportunistic Screening for Carotid Calcifications: A Case-Based Review

**DOI:** 10.7759/cureus.94585

**Published:** 2025-10-14

**Authors:** Guido Schiroli, Pierluigi Valente, Francesco Valente, Lapo Sbrenna, Andrea Sbrenna

**Affiliations:** 1 Dentistry, University of Genoa, Genoa, ITA; 2 Dentistry, Vita-Salute San Raffaele University, Milan, ITA; 3 Oral Implantology, San Damiano Dental Clinic, Rome, ITA; 4 Dentistry, Alma Mater Studiorum University of Bologna, Bologna, ITA; 5 Private Practice, Humanis Dental Center, Perugia, ITA

**Keywords:** carotid artery calcification, carotid artery doppler ultrasound, dental panoramic calcification, dental panoramic radiograph, dental x rays, incidental findings dental panoramic

## Abstract

The incidental detection of carotid artery calcifications on routine panoramic radiographs highlights the preventive role of dentists in identifying patients at cardiovascular risk. This case-based review describes the unexpected finding of unilateral radiopaque masses at the level of the cervical vertebrae C3-C4 on the right side of a panoramic radiograph, consistent with carotid artery calcifications. Such findings are clinically significant due to their possible association with cerebrovascular and cardiovascular events, including stroke and myocardial infarction. Although panoramic radiographs are not designed as screening tools for vascular disease, they may incidentally reveal calcifications in the carotid region, offering a unique opportunity for early recognition and timely medical referral. Emerging evidence suggests that the prevalence of these incidental findings is not negligible, reinforcing the need for increased awareness among dental professionals. While advanced imaging modalities are more accurate in detecting early vascular changes, panoramic radiographs are routinely performed in dental practice, making them a valuable tool for opportunistic detection. This case highlights the importance of interdisciplinary collaboration, where dentists play a pivotal role in the early identification of systemic conditions beyond the oral cavity, potentially reducing the risk of adverse vascular outcomes.

## Introduction

Carotid artery calcifications (CACs) are radiopaque lesions that may occasionally be identified on panoramic radiographs, typically in the region adjacent to the cervical vertebrae C3-C4. Although panoramic imaging is not designed for vascular assessment, incidental detection of CACs has been documented with variable prevalence across dental populations. In a recent study by Janiszewska-Olszowska et al. [1], involving 4,000 dental panoramic radiographs, the anatomical region encompassing the carotid bifurcation was visible in 2,189 images (54.73%). Among these, CACs were identified in 468 cases (21.68%), underscoring the potential diagnostic relevance of such incidental findings in routine dental imaging, particularly when the cervical region is adequately visualized through high-quality acquisition techniques [1]. Similarly, Acikgoz et al. analyzed 9,553 digital panoramic radiographs and found atherosclerotic plaques consistent with CACs in 5.8% of cases [2]. These findings further support the potential of panoramic radiography as a tool for opportunistic detection of vascular calcifications, despite its primary diagnostic focus being limited to maxillofacial structures. The clinical importance of CACs lies in their potential association with atherosclerosis and increased risk of adverse vascular events, including ischemic stroke and myocardial infarction [3,4]. Stroke remains one of the leading causes of morbidity and mortality worldwide, and early identification of at-risk individuals is a major public health priority.

Dental practitioners may play a role in this context, as panoramic radiographs are routinely performed in general dental practice. Recognition of CACs on such images could prompt timely referral for medical evaluation, particularly in patients not previously screened for cardiovascular risk factors [5,6]. However, panoramic radiographs lack specificity, and confirmatory imaging, such as carotid Doppler ultrasonography, remains essential for definitive diagnosis and management. Recent advances in artificial intelligence have shown promise in enhancing the accuracy of CAC detection on dental radiographs, potentially supporting the role of dentists in opportunistic cardiovascular risk assessment; however, these approaches remain limited by potential data bias and false-positive findings and require further external validation [7,8]. Nevertheless, the predictive value of CACs identified on dental imaging for future adverse events remains inconclusive, and further interdisciplinary research is needed to clarify their diagnostic and prognostic implications. This case-based review aims to illustrate the incidental detection of CACs in a dental patient, summarize current evidence regarding their prevalence and clinical significance, and highlight the importance of awareness among oral health professionals.

This case report is based on routine clinical documentation and observational findings and does not constitute research involving human subjects under current institutional and regulatory definitions. In accordance with institutional policy, the case was deemed exempt from formal IRB/Ethics Committee review. Written informed consent was obtained from the patient for the dental treatment and for publication of this report and any accompanying images. The images included do not contain any data that could lead to patient identification.

## Case presentation

**Figure 1:**
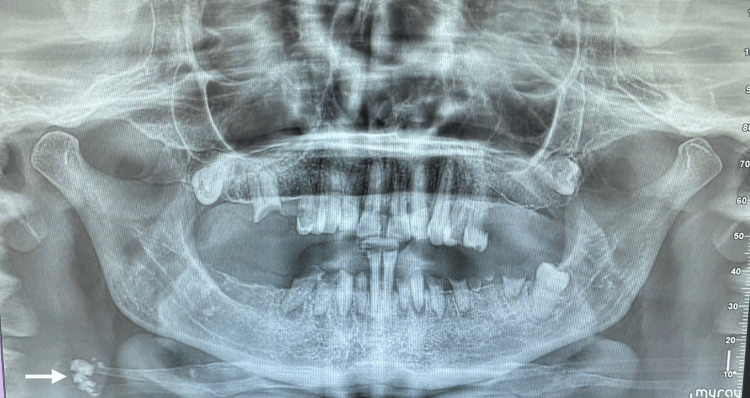
Panoramic radiograph showing radiopaque nodular calcifications in the right carotid region, adjacent to the intervertebral space between C3 and C4 (white arrow), consistent with carotid artery calcifications.

A 58-year-old female patient presented to the San Damiano Dental Clinic in Rome, Italy, for an oral examination and evaluation for potential implant-supported prosthetic rehabilitation. Her medical history was notable for arterial hypertension, and she was undergoing long-term therapy with cardioaspirin. Although she reported no previous cerebrovascular events, she was under neurological evaluation due to significant gait disturbances. As part of the routine dental diagnostic work-up, a panoramic radiograph was obtained. In addition to expected dental and alveolar findings, the image revealed a unilateral radiopaque nodular calcification in the cervical region, adjacent to the intervertebral space between C3 and C4, consistent with CACs (Figure 1).

The patient was referred for further vascular evaluation. A comparative Doppler ultrasonography of the neck vessels was performed using a high-frequency linear transducer (5-10 MHz). The examination revealed bilateral increased intimal echogenicity and diffuse atherosclerotic changes, with intima-media thickness ranging from 0.11 to 0.12 cm (normal <0.07 cm [9]). No abnormal flow velocities were detected along the common carotid arteries.

**Figure 2:**
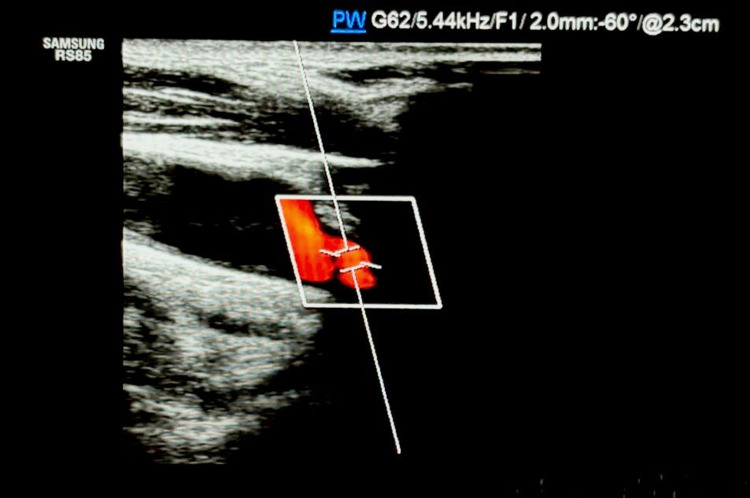
Color Doppler ultrasonography of the right carotid bifurcation demonstrating hyperechoic nodular calcifications, corresponding to the radiopaque lesion previously identified on panoramic radiography.

**Figure 3:**
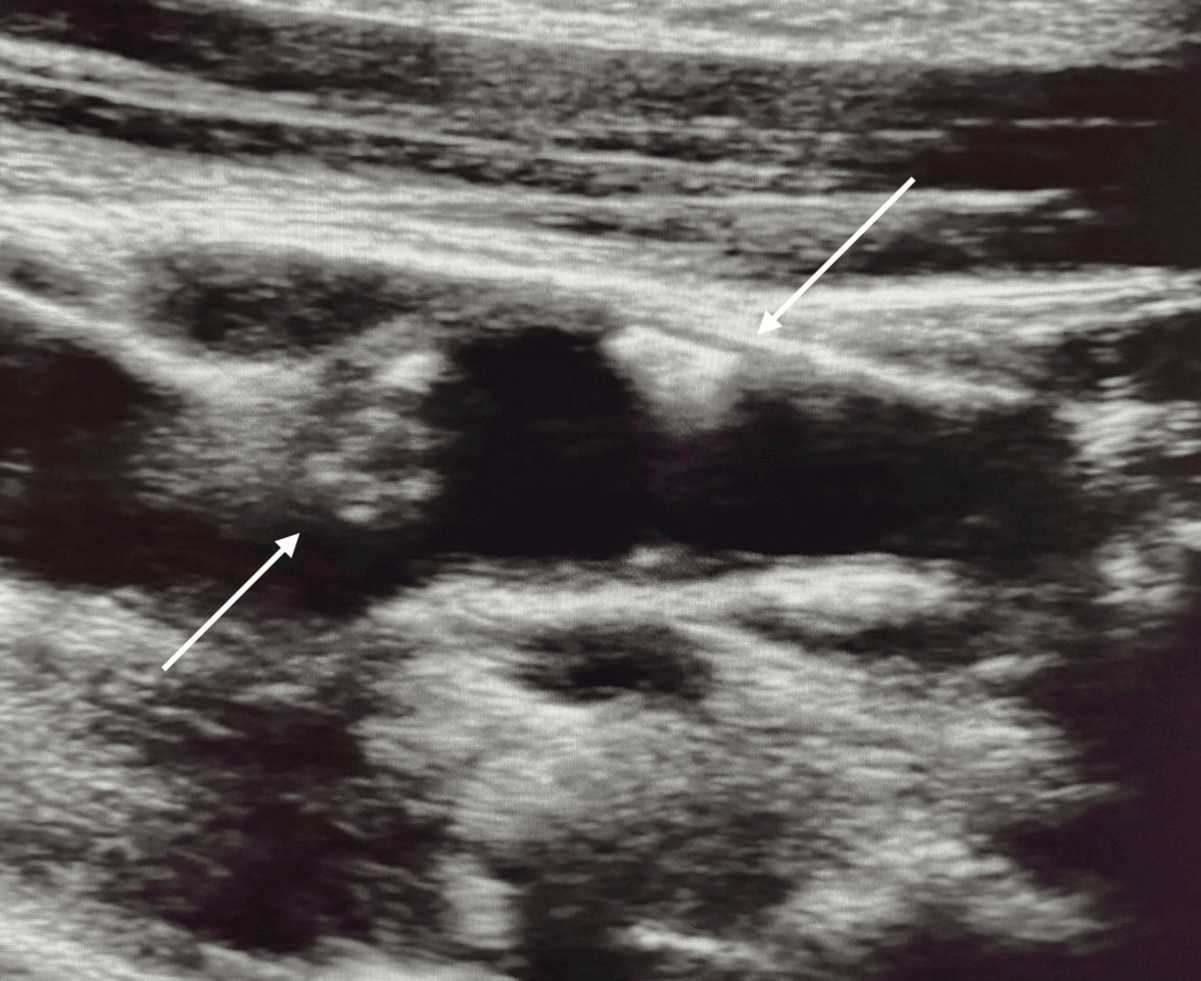
B-mode ultrasound image of the right carotid bifurcation confirming the presence of calcified plaque (white arrows), consistent with the radiopaque lesion observed on panoramic radiography.

**Figure 4:**
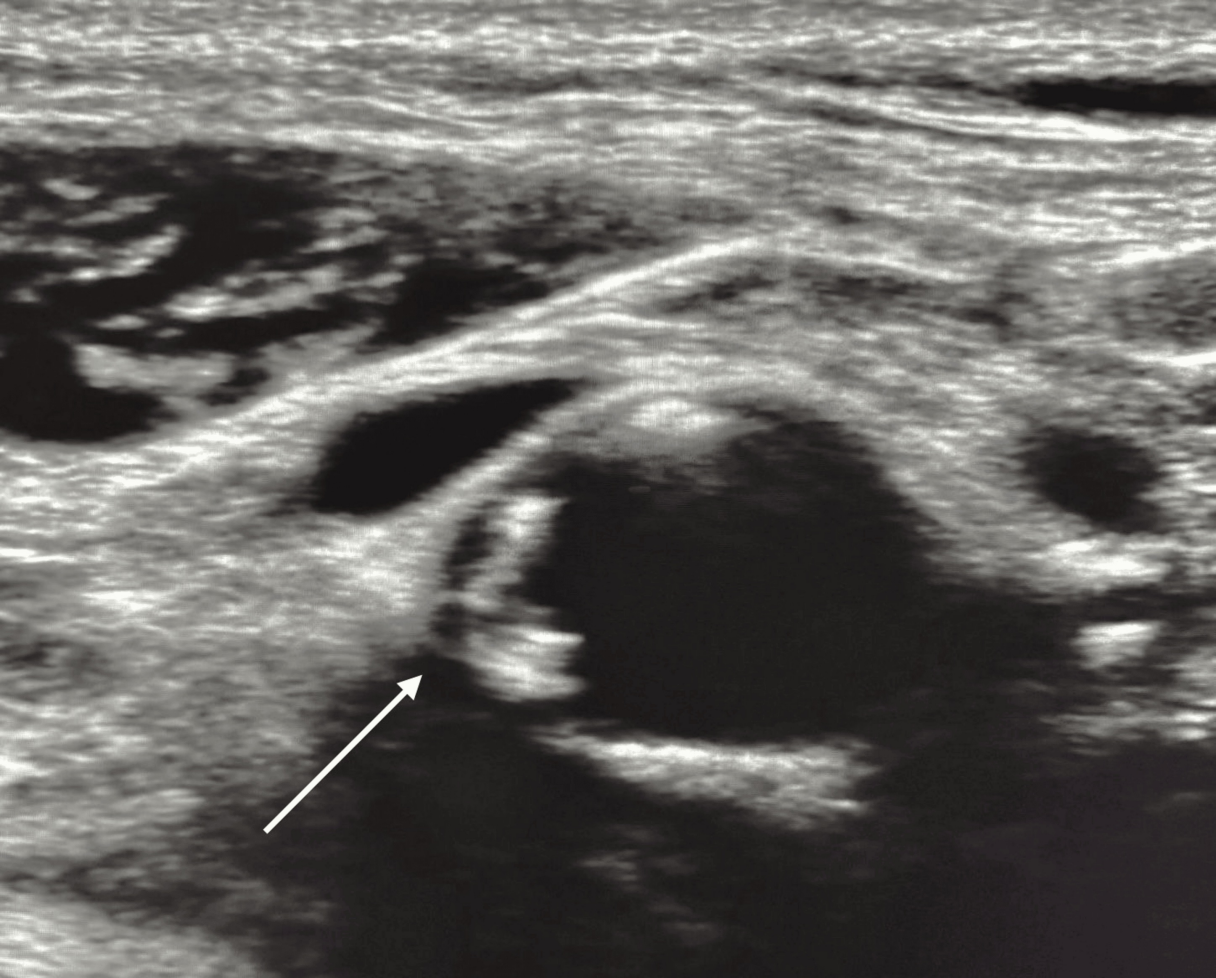
Additional B-mode ultrasound image of the right carotid bifurcation showing a calcified atheromatous plaque (white arrow), corresponding to the radiopaque lesion observed on panoramic radiography.

On the right side, the carotid bulb demonstrated marked intima-media thickening up to 0.13 cm, with concentric fibrocalcific parietal deposits extending into the internal carotid artery (Figures 2-4).

The geometric reduction of the lumen at the carotid bifurcation was estimated at approximately 50%, calculated according to the European Carotid Surgery Trial (ECST) method, and consistent with the criteria outlined by the European Society for Vascular Surgery guidelines [9], without evidence of haemodynamically significant acceleration on pulsed Doppler. The external carotid artery demonstrated a sub-stenosis of approximately 20%, with no associated flow abnormalities.

On the left side, a similar condition was observed, with stenosis of the carotid bulb and proximal internal carotid artery reaching up to 60%. Flow demodulation was noted, although no pathological acceleration was detected. The plaques appeared calcified and stable, extending along the proximal segment of the internal carotid artery. The external carotid artery demonstrated non-significant stenosis (30%) with preserved hemodynamic parameters.

Bilateral vertebral arteries revealed marginal arthrosic changes without evidence of endoluminal stenosis or dominance. Calcifications were also noted at the origin of the subclavian arteries, without significant luminal narrowing. Overall, the findings indicated advanced atherosclerotic involvement of the extracranial vessels supplying the brain. These findings were consistent with advanced yet stable atherosclerotic disease. Although no critical hemodynamic stenosis was detected, the presence of bilateral fibrocalcific plaques and diffuse intima-media thickening warranted interdisciplinary follow-up and comprehensive cardiovascular risk stratification.

## Discussion

The incidental detection of radiopaque foci in the carotid region on panoramic radiography carries important diagnostic and preventive implications for dental clinicians, as well as for the broader management of the patient described. This case, featuring unilateral nodular calcifications at the level of C3-C4, aligns with a well recognized spectrum of incidental findings on orthopantomography that may represent CACs, and, therefore, warrants a cautious, evidence-based response. Epidemiological studies reveal considerable heterogeneity in the reported prevalence of CACs on panoramic radiographs, reflecting variations in population age structure, imaging protocols, and interpretive criteria. Early large-scale studies [10,11] estimated a prevalence of approximately 4-5% in general adult dental populations. In contrast, investigations targeting older cohorts with known vascular disease [12] or those employing panoramic images with broader anatomical coverage have reported significantly higher detection rates. These discrepancies underscore the context-dependent nature of prevalence estimates and reinforce the role of advanced age as a key determinant in CAC identification.

The diagnostic concordance between panoramic radiographic findings and vascular imaging is imperfect yet clinically meaningful. Prospective studies comparing orthopantomograms with carotid duplex ultrasound have demonstrated that a substantial proportion of patients with radiographic evidence of CACs present with clinically significant carotid stenosis. In one matched cohort, the prevalence of ≥50% stenosis was approximately 15.4% among patients with CACs on panoramic radiographs, compared to 5.8% in those without such radiographic signs [13]. In a longitudinal cohort study, Bengtsson et al. reported that CACs identified on baseline panoramic radiographs in patients aged 60-96 years were significantly associated with subsequent stroke and ischemic heart disease over more than a decade of follow-up [14]. Older validation studies have reported reasonable concordance between panoramic radiographic signs and duplex sonography, although sensitivity and specificity vary depending on reader experience and the radiographic characteristics [15]. Importantly, panoramic radiographs cannot reliably quantify stenosis or assess plaque vulnerability, as they are two-dimensional, offer limited soft-tissue contrast, and may yield false positives due to adjacent calcified structures - such as stylohyoid ossification, calcified thyroid cartilage, tonsilloliths, triticeous cartilage, and other head-and-neck soft tissue calcifications. Large retrospective datasets that systematically cataloged these entities have shown that atherosclerotic plaques represent only one of several possible findings visible on panoramic imaging [2]. Accurate anatomical localization, typically near the C3-C4 interspace, and careful morphological assessment can reduce, but not eliminate, diagnostic uncertainty.

Beyond diagnostic concordance, several studies have explored whether CACs identified on dental panoramic radiographs serve as markers of systemic atherosclerotic burden and predictors of adverse vascular outcomes. A systematic review concluded that patients with CACs on panoramic imaging are, in some cohorts, more likely to experience cerebrovascular or cardiovascular events, including stroke, transient ischemic attack, myocardial infarction, and the need for revascularization, although the available evidence remains heterogeneous and not uniformly statistically conclusive [3]. Cohort data from patients undergoing coronary angiography further demonstrated that CACs detected on panoramic radiographs correlated with markers of coronary atherosclerosis and were associated with higher coronary calcium scores [16]. Longitudinal population studies have linked radiographic CACs with increased all-cause and cardiovascular mortality, and severe CACs observed on panoramic imaging have been associated with reduced survival rates [17]. Smaller, condition-specific series have also reported significant associations between CAC presence and traditional vascular risk factors such as hypertension and prior myocardial infarction, although some of these findings are based on limited sample sizes and should be interpreted with caution [12]. Collectively, these data support the notion that CACs incidentally identified on dental radiographs may reflect underlying systemic atherosclerosis and an elevated vascular risk profile in selected patients. However, they do not justify the use of panoramic imaging as a population-level screening tool for carotid artery disease.

The interrelationship between oral infection, systemic inflammatory burden, and vascular calcification has been investigated in several studies. In the Parogene cohort, a Finnish sub-cohort of the larger Corogene study, consisting of approximately 500 patients undergoing coronary angiography with subsequent standardized dental and radiographic examinations, CACs detected on panoramic imaging were associated with markers of dental infection and dysbiotic subgingival bacterial profiles, while severe CACs predicted increased cardiovascular mortality [17]. Historical research has also demonstrated strong associations between indicators of periodontitis and positive carotid duplex findings [15]. These observations are biologically plausible and align with the paradigm that chronic oral inflammation may contribute to systemic inflammation and atherogenesis. However, they do not establish a direct causal link between a single dental lesion and cerebrovascular events and should therefore be interpreted with caution.

From a clinical management perspective, published reviews and consensus recommendations support a pragmatic approach. When a dentist identifies a radiographic appearance suggestive of CACs, the finding should be documented in the patient’s record, communicated clearly yet non-alarmingly, and followed by a recommendation for medical evaluation to assess cardiovascular risk [5,6]. Confirmatory imaging, most commonly carotid duplex ultrasonography, is necessary to determine the presence and severity of stenosis and to guide further management. The decision to pursue advanced imaging modalities (e.g., CT angiography, coronary calcium scoring) should be individualized, based on clinical risk assessment and local cardiovascular care pathways. Notably, some studies have shown that patients with CACs on panoramic radiographs exhibit a significantly higher prevalence of elevated coronary calcium scores, suggesting that incidental detection may help identify individuals who could benefit from broader cardiovascular evaluation [5-13]. However, not all patients with CACs on panoramic imaging require immediate invasive investigation. Lim et al.’s systematic review [3] highlighted that individuals already under active management for atherosclerotic disease may derive limited additional benefit from routine referral triggered solely by a dental radiographic finding. This underscores the importance of contextualizing CAC detection within the broader clinical picture.

Recent advances in automated image analysis hold promise for improving detection rates and standardizing reporting. Deep convolutional neural networks and transformer-based models applied to large panoramic datasets have demonstrated high sensitivity and specificity in research settings [18,19], and a recent meta-analysis reported pooled performance metrics that are encouraging for potential screening applications [7]. These technologies may enhance clinician performance and reduce missed findings, but they require external validation across diverse populations, seamless integration into clinical workflows, and careful consideration of false positives and the medico-legal implications of automated alerts. Until such tools are broadly validated and regulated, human oversight and diagnostic confirmation remain essential.

The limitations of the current evidence base and practical considerations warrant emphasis. Panoramic radiography is neither designed nor validated as a diagnostic tool for vascular disease. Its two-dimensional projection, intersubject anatomical variability, and the presence of multiple benign calcified structures constrain both specificity and sensitivity. Prevalence and prognostic studies differ in design, sample composition, and outcome definitions, resulting in heterogeneity that complicates causal inference. Moreover, several cohorts include older individuals or patients with established cardiovascular disease, inflating event rates and limiting generalizability to younger, lower-risk dental populations [1-17]. Finally, over-referral based on uncertain radiographic impressions may incur logistic, economic, and psychological burdens; a balanced, patient-centered approach is therefore recommended.

Based on current evidence and recommended practice, pragmatic steps following the incidental detection described in this case include the following: (a) accurate documentation of the radiographic finding, including laterality and anatomical position relative to the C3-C4 interspace; (b) clear, non-alarming communication to the patient regarding the uncertain yet potentially significant nature of the finding; (c) referral to the patient’s primary care physician or cardiology service for cardiovascular risk assessment and consideration of carotid duplex ultrasonography; and (d) coordination with the medical team to ensure that further investigations and management strategies are tailored to the patient’s overall risk profile and comorbidities [5,6-13].

The dentist’s role in this context is primarily one of opportunistic detection and timely communication, rather than definitive vascular diagnosis. Panoramic radiographs may incidentally reveal calcifications in the carotid region that, in many cohorts, are associated with systemic atherosclerotic disease and an increased risk of cerebrovascular events. However, panoramic imaging should be considered an opportunistic prompt for medical evaluation rather than a definitive diagnostic tool. This case underscores the importance for dental practitioners to recognize the radiographic features of CACs, to document and communicate such findings responsibly, and to collaborate with medical colleagues for confirmatory imaging and appropriate cardiovascular risk management.

## Conclusions

This case highlights the incidental discovery of CACs on a routine panoramic radiograph, later confirmed by ultrasonography. While not intended for vascular diagnosis, panoramic imaging’s widespread use in dentistry offers a chance to identify significant findings early. Timely referral for medical evaluation may aid stroke prevention, underscoring the dentist’s role in broader health surveillance. Future research should focus on standardizing radiographic reporting criteria, prospectively validating automated detection algorithms across diverse populations, and clarifying which patient subgroups identified in dental settings derive measurable benefit from expedited vascular investigations.
